# Infantile Rosai-Dorfman Disease With Isolated Brain Lesions Disseminated to the Parenchyma and Intraventricular Ependyma, Alteration of Leukocytes as a Promotion Factor in Immune Defense, and New Proposals: A Case Report and Literature Review

**DOI:** 10.7759/cureus.52453

**Published:** 2024-01-17

**Authors:** Nasser Kamalian, Shahmir Kamalian, Mohammad Vasei

**Affiliations:** 1 Pathology, Shariati Hospital/Tehran University of Medical Sciences, Tehran, IRN; 2 Radiology, Massachusetts General Hospital, Boston, USA; 3 Cell-Based Therapies Research Center, Shariati Hospital/Tehran University of Medical Sciences, Tehran, IRN

**Keywords:** intraventricular ependyma, brain parenchyma, brain, rosai-dorfman disease, infantile

## Abstract

The patient is a one-year-old girl referred to the hospital for an enlarged head after a 1.5-month history of two falls, followed by polydipsia, polyuria, and slow movement and growth. Three subsequent magnetic resonance imaging (MRI) examinations of the brain revealed nodular lesions disseminated in the brain parenchyma and intraventricular ependyma, resulting in obstructive hydrocephalus. Thoracic and abdominopelvic sonography showed no additional lesions. The preliminary diagnosis was a primary or metastatic neoplasm or infection. A biopsy of a lesion in the right frontal lobe was taken. The histological examination revealed features of Rosai-Dorfman disease (RDD), consisting of limited perivascular lymphoplasma cell infiltration with intervening sheets of proliferated histiocytes, with some of the histiocytes showing endocytosis of a single intact lymphocyte (emperipolesis).

## Introduction

Rosai-Dorfman disease (RDD) is a non-Langerhans cell histiocytosis (LCH) first described in 1965 by Destombes in four African children with massive lymphadenopathy and termed it "adenitis with excess lipids" [1]. In 1969, Rosai and Dorfman reported a separate series of four young black males [2]. They had characteristic painless bilateral cervical lymphadenopathy with B-symptoms, elevated erythrocyte sedimentation rate (ESR), anemia, and polyclonal gammopathy. The histomorphology of the lesion consists of proliferated histiocytes mixed with lymphoplasma cells within the wide sinuses of enlarged lymph nodes (size ≥7 cm). Hence, it was named as sinus histiocytosis with massive lymphadenopathy [2]. Subsequent studies have shown that extranodal involvement is more common than nodal disease, with skin involvement being the most frequent site. Central nervous system (CNS) involvement, on the other hand, is rare [3,4]. Mortality is seven percent due to complications of infection or amyloidosis [5].

In 1990, Foucar, Rosai, and Dorfman also reviewed 423 cases of nodal and extranodal lesions and reported them to the international registry [5]. In 2020, authors at Mayo Clinic studied the summary of this report and the history of 64 additional patients referred to their tertiary center between 1994 and 2017. The patients' ages ranged from two to 79 years, with a median age of 50. The female-to-male ratio was 1.5:1. The disease was classified into familial, classical (nodal only), extranodal, neoplastic-associated, and immune and Immunoglobulin G4-related (IgG4-related) types. Kirsten rat sarcoma virus (KRAS) and mitogen-activated protein kinase kinase 1 (MAP2K1) mutations were identified in one-third of the cases, suggesting that RDD is neoplastic in this subset of patients. All tissues and organs can be involved. Classical nodal involvement was observed in eight percent of cases, extranodal only in 67%, and non-cervical nodal involvement in 25%. Skin lesions were present in 52% of cases, while CNS lesions were observed in eight percent and were typically parenchymal and dural-based. Although historically considered a benign entity, RDD exhibits diverse manifestations and outcomes. The mortality rate is two percent. The diagnosis typically requires multiple biopsies, with a median of two (range 1-6). The size of lymph nodes ranged from one to two centimeters [6]. Here, we presented a new presentation of Rosai-Dorfman disease in an infant with isolated brain lesions disseminated to the parenchyma and intraventricular ependyma.

## Case presentation

The patient was a one-year-old girl referred to the hospital for an enlarged head. She was healthy until the age of 10.5 months, when the parents noticed that the child had experienced two falls, increased thirst, and urination, along with slow movements and growth.

The child was the product of a 38-year-old mother who underwent in vitro fertilization (IVF) and cesarean section for delivery following six years of infertility. The mother had a history of salpingitis and hydrosalpinx, and she received treatment with metformin for polycystic ovaries. Her pregnancy was full-term and supported by estradiol and progesterone. The father, a healthy 36-year-old, had a normal spermatogram. There was no remarkable family history.

Significant laboratory findings included a serum D-dimer level of 1026 ng (normal: up to 443 ng), cerebrospinal fluid (CSF) protein level of 80 CUmm (normal: 14-50 CUmm) and CSF glucose level of 53 mg/dl (normal: 60-80 mg/dl). CSF polymerase chain reaction (PCR) detected the presence of nontuberculous (non-TB) mycobacterium and Aspergillus. Other tests such as complete blood count (CBC), ESR, C-reactive protein (CRP), serum immunoglobulin G (IgG), immunoglobulin M (IgM), and immunoglobulin A (IgA) levels, CSF culture, and CSF PCR for viral infections and toxoplasma were negative. CSF cytology was negative for neoplastic cells.

**Figure 1:**
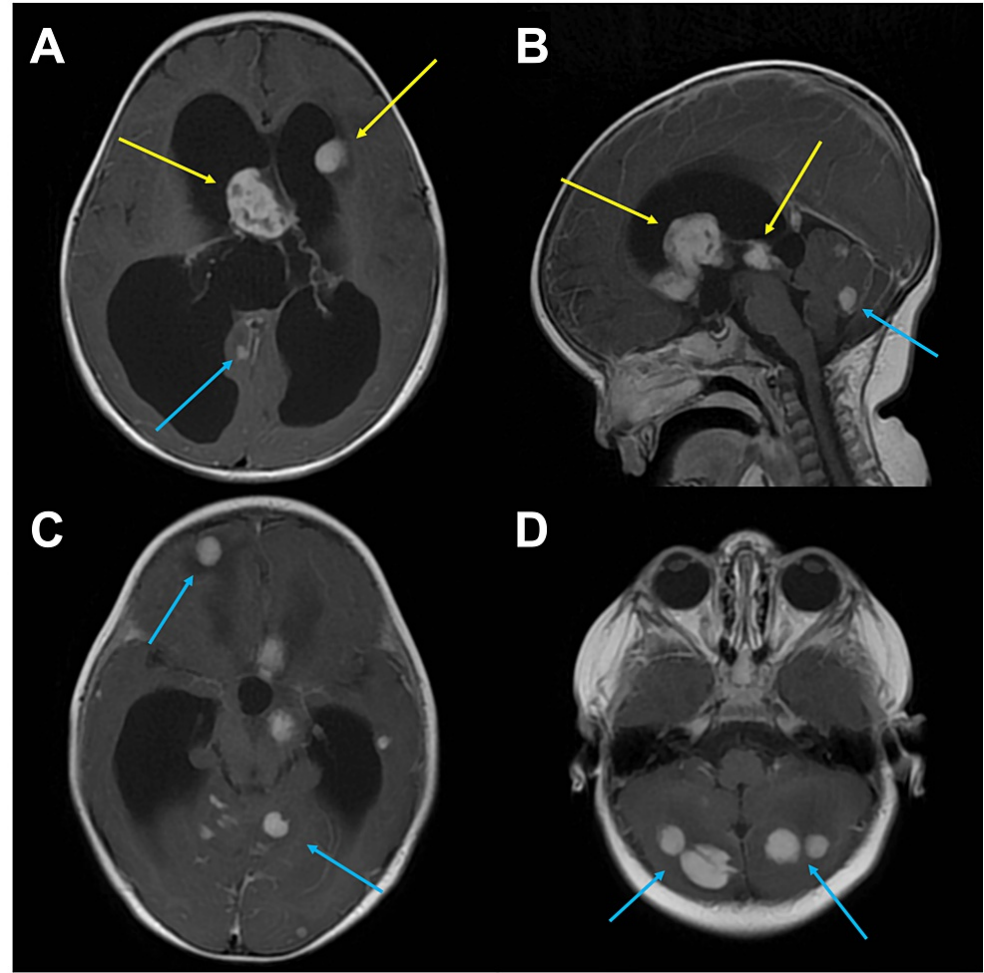
Initial Brain MRI at one year of age The axial (A) and sagittal (B) contrast-enhanced T1-weighted images depict lesions arising from the ventricular lining (yellow arrows), causing noncommunicating hydrocephalus due to the obstruction of the aqueduct of Sylvius. In addition, two additional axial images (C and D), also contrast-enhanced T1-weighted, reveal multiple enhancing brain parenchymal lesions within the supratentorial and infratentorial compartments (indicated by blue arrows).

The initial brain MRI, performed at the age of one year, revealed at least three enhancing lesions with restricted diffusion arising from the ventricular lining, measuring up to 26x28 mm, causing noncommunicating hydrocephalus. Multiple additional intra-axial enhancing lesions, measuring up to 18 mm, were observed in the cerebral hemispheres, cerebellar hemispheres, and brainstem (Figure 1).

**Figure 2:**
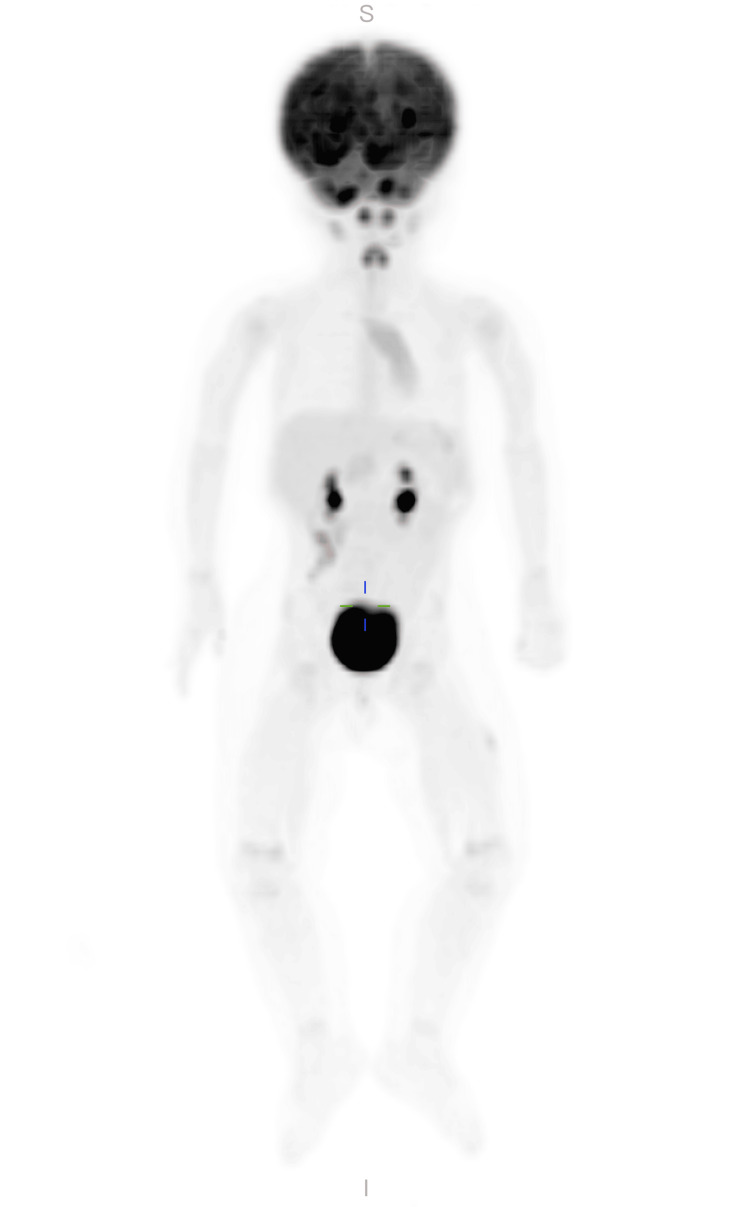
Whole body PET scan shows no evidence of extracranial disease

A repeat MRI, performed two months later, revealed postsurgical changes related to the biopsy of a right frontal lesion and insertion of a right frontal approach ventriculoperitoneal shunt with a thin extra-axial collection over the right cerebral hemisphere, multiple unchanged enhancing masses distributed in the bilateral frontal lobes, right occipital lobes, thalamus, brainstem, and cerebellum, and multiple unchanged enhancing intraventricular lesions. An additional follow-up brain MRI and total spine MRI without contrast, performed 1.5 months later, once again revealed a thin extra-axial collection over the right frontal lobe, indicating a post-operation hygroma. The shunt tip was observed in the lateral ventricle, and hydrocephalus had resolved. Similar to the previous MRIs, multiple abnormally enhancing intra-axial masses were present in the supratentorial and infratentorial compartments. These masses were surrounded by significant edema and showed relative diffusion restriction, particularly in the lesion of the septum pellucidum. No abnormality or evidence of seeding was found on the total spine MRI. Subsequently, a positron emission tomography (PET) scan was performed, which yielded negative results for extracranial disease, confirming the isolation of the disease to the CNS (Figure 2).

**Figure 3:**
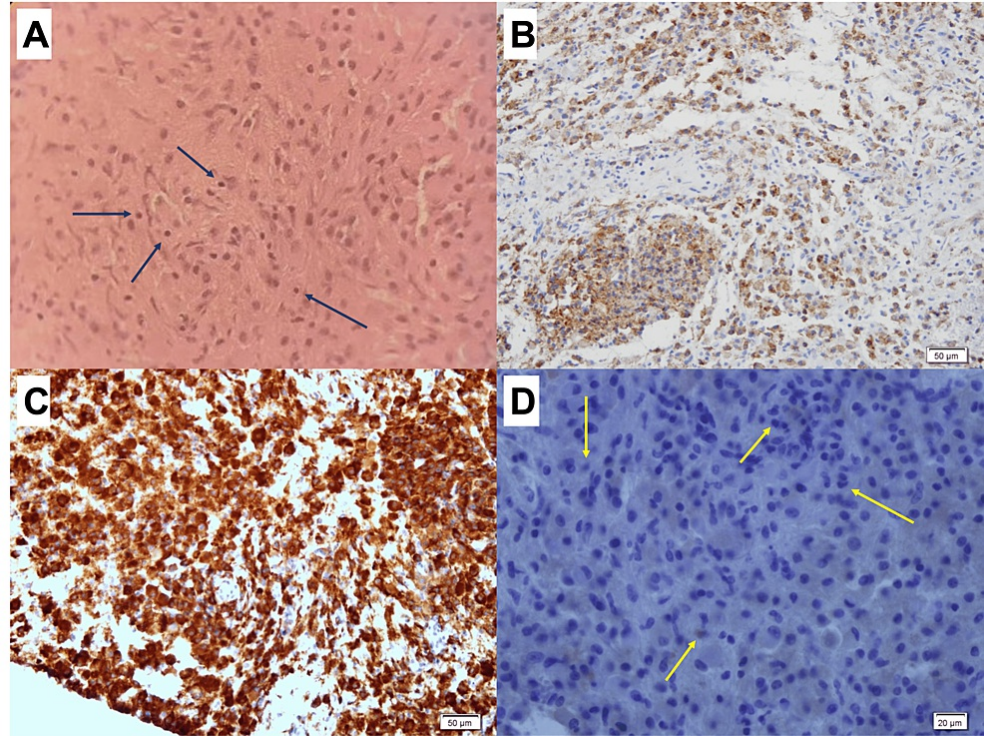
RDD in brain lesion biopsy (A) Hematoxylin and eosin (H&E) stain, 100X magnification, showing sheets of monotonous histiocytes with a moderate amount of dense cytoplasm with intaking individual small lymphocytes with peripheral halos (navy arrows). (B) and (C), both 200X magnifications, histiocytes are highlighted by immunohistochemistry for CD 68 and CD163, respectively. (D) 200X magnification, CD1a is negative and it is seen only in a few cells (yellow arrows). RDD - Rosai-Dorfman disease

Histological examination revealed sheets of histiocytes with mild lymphoplasma cell infiltration, limited to the perivascular area. The histiocytes had round to ovoid small to medium-sized eccentric nuclei and a moderate amount of dense periodic acid-Schiff (PAS)-positive cytoplasm. Scattered histiocytes were identified, each containing a single intact lymphocyte with a peripheral halo in the cytoplasm (emperipolesis, Figure 3A). PAS and Ziehl-Neelsen staining were negative for acid-fast bacilli and fungi. The immunohistochemistry (IHC) panel showed positive results for CD68, CD163, vimentin, integrase Interactor 1 (INI-1), CD1a in a few cells, and Ki67 in 10% of histiocytes, while being negative for oligodendrocyte transcription factor 2 (Olig2), CD56, cytokeratin, myeloperoxidase (MPO), and CD34 (Figures 3B, C, and D).

The previous institutions provided the following diagnoses: 1) diffuse histiocytic proliferation with mild chronic inflammation and 2) chronic meningoencephalitis with xanthomatous macrophagic infiltration. However, at our neuropathology center, positive staining for S100 in histiocytes supported the diagnosis of RDD.

The outcome was favorable as the child remained symptom-free without treatment, with a slight 3 cm growth deficit compared to the average for her age. A follow-up MRI, 15 months after the biopsy and shunt insertion, showed no changes in the size and number of the lesions.

## Discussion

RDD, also known as sinus histiocytosis with massive lymphadenopathy, is a variant of histiocytosis that represents pathological evidence of lymphoplasma cell infiltration and reaction in the background, accompanied by the proliferation of histiocytes showing the diagnostic feature of endocytosis of intact lymphocytes (emperipolesis). This inflammatory proliferative reaction serves as an innate defense mechanism against a type of lymphocytes recognized by the host tissue as foreign or altered cells. The etiology of RDD remains unknown, although bacterial and viral factors (e.g., human herpesvirus 6 [HHV6] and Epstein-Barr virus [EBV]), as well as immune disorders, are suggested to act as triggering factors [7]. Familial, neoplastic-associated, and IGG-4-related cases have also been described. Furthermore, the presence of KRAS and MAP2K1 mutations in one-third of patients supports a neoplastic process. Endocytosis of other leukocytes has been identified in various host cells [8]. It seems that these immune cells act as mediators to suppress the pathogenicity of injurious agents, and the different clinical forms and outcomes of disease entities are related to the rate of immunocompetency of these cells. In RDD, lymphocytes migrate into the host tissue for localized defense. They capture antigens through their surface receptor molecules and undergo alterations, thereby functioning as suppressor cells against injurious agents.

We identified 18 cases of isolated RDD with CNS involvement in the literature. These cases included four pediatric patients [9-12] and 14 adult patients [7,13-15], which are summarized in Table 1.

**Table 1:** Summarizing four pediatric and 14 adults of isolated CNS RDD CNS - central nervous system; RDD - Rosai-Dorfman disease; GBM - glioblastoma

Author, year, reference	Case no	Age (y)	Sex	Presentation	Location	Preoperative diagnosis	Treatment	Outcome
Shaver, et al. 1993 [9]	1	5	M	Progressive ptosis of the left eye developed one month after chickenpox	Cavernous sinus	Meningioma or trigeminal schwannoma	Surgery and steroid	Disease-free at one month
Di Rocco, et al. 2003 [10]	2	13	F	Headache, vomiting, fever	Left frontal lobe	Not reported	Surgery twice	Recurrent disease at three months, disease-free at 12 months
Woodcock, et al. 1999 [11]	3	15	F	Headache, amenorrhea, blurred vision	Suprasellar	Not reported	Steroid	Slight increase in size of the lesion at nine months
Griffiths, et al. 2004 [12]	4	9	M	Headache, seizures	Right frontal	Meningioma	Total resection	Disease-free after 43 months
Andriko, et al. 2001 [13]	5-15	22-63	7M-4F	Headache, seizures, numbness, paraplegia	Eight intracranial, three spinal	Meningioma in seven out of eight patients with intracranial disease. Not reported for other cases.	Surgical	One patient died five days after surgery due to surgical complications. Mean follow-up at 15 months showed no recurrence after total resection and no clinical or radiographic progression of disease in patients with incomplete resection.
Fukushima, et al. 2011 [14]	16	33	F	Headache	Left frontal lobe	Primary brain tumor, metastatic disease or lymphoma	Surgical, prednisolone	Disease-free at five months
Mahzoni, et al. 2012 [15]	17	33	M	Headache, ataxia, unconsciousness	Left temporal lobe	GBM or lymphoma	Surgical	Reported as alive at 14 months
Li, et al. 2021 [7]	18	26	F	Two years history of fatigue, joint pain, nausea, vomiting, polydipsia, polyuria and 20 lbs weight gain	Hypothalamus, disseminated craniospinal leptomeningeal nodules and systemic lymphadenopathy	Not reported	High dose of steroids and chemotherapy	Not reported

RDD is related to LCH, as cytogenetic assays have shown the presence of mitogen-activated protein kinase (MAPK) mutations in both diseases. In LCH, the histiocytes are positive for S-100, langerin, and CD1a, and they contain Birbeck granules (BG). Langerin (CD207) is also found within the structure of BG [16], indicating that it is a cellular residue of CD1a-altered lymphocytes produced through early lytic (macrophagic) reactions of Langerhans cells (LHCs). Therefore, RDD and related conditions such as LCH, Erdheim-Chester disease (ECD) and BG-containing reticulohistiocytoma variant are conditions mediated by the same transfective altered lymphocyte. Therefore, we propose naming these conditions as lymphoid transfective diseases (LTDS).

LHCs are located in the epidermis, and epithelia serve as sentinel cells. They highly express CD1a, and their surface receptors capture glycolipid antigens produced as a result of damage from injurious agents. Subsequently, these antigen-presenting cells migrate into the dermis, where the CD1a-antigen complex is recognized by CD4 T-cells. CD4 T-cells, along with their variant CDH22 T-cells, actively secrete interleukin 22 and gamma interferon, which are essential components for skin defense and repair [17-20]. According to researchers, a significant percentage (1 in 50) of activated CD4 T-cells can be found in the bloodstream, and through their surface chemokine receptors, they are able to migrate into various tissues [19]. This phenomenon forms the basis for the recurrence of the disease, either earlier or later, when the immunity of the primary or other host tissues is compromised. We observed a decreased immune response in the reported summary of five adult and five pediatric patients (aged 2-15 years) who developed CNS RDD disease following cervical and extranodal involvement, with an interval of 0-20 years [9].

The histologic diagnosis of RDD is challenging. The classic histopathologic features are enlarged histiocytes, at times multinucleated, with large vesicular nuclei and abundant pale waxy or eosinophilic glassy cytoplasm, showing emperipolesis. However, in certain extranodal lesions, severe inflammatory background and/or fibrosis can obscure the diagnostic feature of emperipolesis, leading to misdiagnosis as chronic inflammation. In these difficult cases, establishing the correct diagnosis often required multiple biopsies, with a median number of two (range: 1-6). In our patient, the pathologic features of the lesion were somewhat different from those of classical nodal RDD. The histiocytes showed rather uniform small to medium-sized round to ovoid hyperchromatic nuclei and a lesser amount of dense, PAS-positive cytoplasm. In contrast to classic RDD, the inflammatory background was less prominent, mostly limited to perivascular spaces, and emperipolesis was easily identified.

Emperipolesis is defined as the presence of a living cell within another living cell [21]. Although it is required for the diagnosis of RDD, it is not specific to this condition. Emperipolesis can be observed in various physiological and pathological conditions, including sarcoidosis [22], LCH [23], ECD [24], Hodgkin and non-Hodgkin lymphoma [25,26], neuroblastoma [21,27], epithelioid glioblastoma [28], and others [21].

When interpreting pathologic specimens, it is crucial to ensure that the tissue obtained through needle biopsy, fine-needle aspiration (FNA), and imprint cytology is adequate. Additionally, careful attention should be given to the accompanying cells and other suspicious changes to differentiate between a malignant condition and RDD, particularly in patients presenting with lymphadenopathy and B-symptoms [8].

Management and treatment of RDD should be individualized based on the number of lesions and organs involved, symptoms, and disease recurrence [29]. In this regard, based on our clinicopathological view, we propose to categorize RDD lesions into a spectrum, which is benign on one side, recurrent and less aggressive (intermediate), and neoplastic or systemic on the other side.

The recommended treatment approach for RDD, as advised by the National Comprehensive Cancer Network (NCCN), includes the following: 1) surgical intervention for unifocal organ involvement and patients with spinal cord compression; 2) external beam radiation for localized unresectable masses; 3) steroid therapy for bone, orbits, and CNS involvement without complete response; 4) sirolimus, which has shown benefits for RDD in children; 5) chemotherapy with rituximab and cladribine for cases of massive lymphadenopathy and refractory disease; 6) immunotherapy with tumor necrosis factor-α (TNF-α) inhibitors, thalidomide, and lenalidomide for patients with increased levels of TNF-α and interleukin 6 (IL-6), as well as refractory nodal and bone disease; 7) targeted therapy with imatinib may be an option for cases of systemic involvement [30].

The prognosis is generally favorable for cutaneous, nodal, and osseous forms of the disease. Most patients with CNS involvement also have a favorable prognosis, although in some cases, it can progress and have a fatal course. Additionally, approximately 40% of cutaneous and nodal lesions undergo spontaneous regression [30].

From a clinicopathological perspective, RDD and its related conditions show a predilection for immune-deficient individuals, both young and old. Therefore, beyond surgery for unifocal single lesions [6,29], we propose strategies directed at enhancing local tissue and systemic immunity. These strategies may include vaccination against specific antigens, administration of interleukin 22, interferon-gamma, and immune cell and embryonic stem cell therapies, which need further investigation and experimental evidence to establish their potential benefits and effectiveness.

Our patient initially presented with falls, polydipsia, polyuria, slowness of movements, and growth abnormalities, followed by the development of hydrocephalus. Laboratory tests detected non-TB mycobacterium and aspergillus through a positive molecular panel. CSF cytology showed no abnormalities except for increased protein levels and slightly decreased glucose. Radiological examination revealed diffuse brain parenchymal and intraventricular subependymal lesions. Fifteen months after biopsy and shunt insertion, a follow-up MRI showed no changes in the size and number of lesions, and the patient remains asymptomatic and otherwise well. To the best of our knowledge, no infantile case of RDD has been described in the literature. RDD lacks specific clinical, radiological, and laboratory tests for diagnosis, and the definite diagnosis relies on histomorphology and immunohistochemistry [6]. In this context, our objectives were to increase familiarity with the diagnosis of the disease and report this unique case of infantile RDD. Additionally, we conducted a comprehensive literature review to gain deeper insights into the disease and the effect of the immune defense. It is crucial as the disease's pathogenesis is closely linked to the capacity of leukocytes to bind antigenic lipid molecules via their surface receptors. This binding results in the alteration of leukocytes and reduces the presentation of endogenous lipids in host tissues, ultimately alleviating tissue damage. Therefore, the alteration of leukocytes is a promotion factor in immune defense.

## Conclusions

We reported a unique case of infantile RDD with isolated brain lesions disseminated in the parenchyma and interventricular ependymal lining. The histomorphology of this case was somewhat different compared to the classic nodal form of the disease. In addition, we have suggested that the alteration of leukocytes serves as a promotion factor in immune defense.
